# Eye-Related Factors That Can Be Associated With the Plane of Phacoemulsification

**DOI:** 10.7759/cureus.24578

**Published:** 2022-04-29

**Authors:** Enes Uyar

**Affiliations:** 1 Department of Ophthalmology, Aksaray University, Aksaray, TUR; 2 Ophthalmology, Aksaray University Training and Research Hospital, Aksaray, TUR

**Keywords:** phaco-chop, plane of phacoemulsification, phacoemulsification surgery, nuclear sclerosis, anterior chamber depth

## Abstract

Purpose: To evaluate the effect of eye-related factors such as biometric and surgical parameters, nuclear sclerosis (NS) grade, and pupil and capsulorhexis diameters on the plane of phacoemulsification (PP).

Material and Methods: This prospective study included 328 eyes of 328 patients who underwent phacoemulsification surgery. The phaco-chop technique was performed in all patients and changes in PP that occurred during surgery were recorded. Patients were grouped as follows: Group 1, > 75% of lens nucleus emulsified in the capsular bag; Group 2, > 75% of lens nucleus emulsified at the pupillary plane; and Group 3, > 50% of lens nucleus emulsified in the anterior chamber. The association between PP and eye-related factors was evaluated.

Results: There were 153 patients (46.7 %) in Group 1, 104 patients (31.7 %) in Group 2 and 71 patients (21.6 %) in Group 3. The factors associated with PP were anterior chamber depth (ACD) (p = 0.020) and NS grade (p = 0.028). No significant relationship was detected between PP and age, surgical parameters or other biometric values. Moreover, PP was found to be more anterior in patients with soft cataracts and deeper ACD values (p values were 0.002 and 0.036, respectively).

Conclusion: The present study has reported that PP may move to more anterior, as softer cataracts may increase the fear of posterior capsule rent. Moreover, PP may move to more posterior due to shallow anterior chambers or high-grade cataracts that could potentially increase the fear of endothelial injury.

## Introduction

Phacoemulsification (PP) surgery has become the routine treatment method for cataracts [1]. Basically, this surgery is based on the fragmentation of the sclerotic lens with various techniques and the emulsification of pieces of the lens with ultrasound energy. Ultrasound energy used during the emulsification of lens materials is one of the most important factors affecting corneal edema, which delays visual recovery after surgery [2,3]. Therefore, several strategies are applied to reduce the effect of ultrasound energy on the corneal endothelium [3,4]. Nuclear fragmentation techniques are part of the intraoperative strategies. For example, retro-chop, tilt and tumble and supracapsular half-moon techniques are strategies that are applied at more anterior planes, whereas divide and conquer, stop and chop, and phaco-chop techniques are strategies in which the emulsification happens posterior to the pupillary plane [2,5,6].

The common purpose of several chopping techniques is to optimize the used energy during phacoemulsification and reduce the total operation time [6]. In addition to this purpose, it is also aimed to perform phacoemulsification in the capsular bag and away from the corneal endothelium as much as possible, especially in hard cataracts [6,7]. However, this is not always possible, because phacoemulsification surgery is a dynamic course that is generally performed under topical anesthesia and within a living part of the body. Therefore, the plane at which the emulsification of the lens fragments happens can change during surgery, although the same chopping technique is used.

Several factors may lead to changes in the plane of PP during surgery. These include intraoperative pupil changes, anterior chamber dynamics, prolapse of the lens to the anterior chamber during hydrodissection, floating of the nuclear fragments anterior to the capsular bag, catch of the fragments in the anterior chamber by the ocular viscoelastic device (OVD), avoiding posterior capsule rupture, surgeon comfort and especially surgeon experience. As it is difficult to measure these factors objectively, we focused on several measurable factors such as biometric values, surgical parameters, preoperative pupil diameter with full pharmacological dilatation, and nuclear sclerosis (NS) grade. Thus, the current study aimed to evaluate the association between some eye-related factors and PP. To the best of our knowledge, no study on changes in PP during surgery has been published so far.

## Materials and methods

This prospective study was performed on 341 consecutive patients who were operated on in the Ophthalmology Clinic of Aksaray Training and Research Hospital between November 1, 2019, and November 1, 2021. Informed consent was obtained from all participants. The study was conducted with the approval of the Aksaray University Ethical Committee in accordance with the Helsinki Declaration (Protocol number: 2019/10-02).

All patients underwent complete ophthalmologic examinations preoperatively, including best corrected visual acuity (BCVA) assessment, slit-lamp and fundus examinations, and intraocular pressure (IOP) measurement by a pneumotonometer (TX-20, Canon Inc., Tokyo, Japan). Nuclear opacification (NO) grade was classified clinically according to the Lens Opacities Classification System 3 (LOCS3). Biometric parameters; anterior chamber depth (ACD), axial length (AL), central corneal thickness (CCT), lens thickness (LT), corneal curvature, keratometry value, and pupil diameter measurements after full dilatation were evaluated using low-coherence optical biometry device (Haag-Strait Diagnostics Biometer LS-900; Haag-Strait AG, Switzerland). BCVA, IOP, slit-lamp and fundus examinations were repeated after surgery on day 1, 7, and 30.

The inclusion criteria for this study were as follows: NO2, NO3, NO4, or NO5 grade senile cataract, age above 40 years, not having an acute or chronic eye disease (glaucoma, uveitis, diabetic retinopathy, etc.) and no previous ophthalmological surgery history. The exclusion criteria were as follows: preexisting corneal pathology, having intumescent cataract, pseudoexfoliation syndrome, intraoperative floppy iris syndrome, using iris hooks during surgery because of a small pupil and any eye pathology preventing preoperative and postoperative measurements. In addition, patients who developed intraoperative complications were also excluded from the statistical analysis.

All the surgeries were performed by the same surgeon, using the same phacoemulsification machine (OS4, Oertli Instrumente AG, Berneck, Switzerland) under topical anesthesia and sterile conditions. Pupil dilatation was achieved preoperatively. Two paracenteses, a sodium hyaluronate 2.0% (Protectalon; VSY Biotechnology GmbH, Leinfelden-Echterdingen, Germany) OVD injection into the anterior chamber and a superior clear corneal main incision using a 2.75 mm blade, respectively, were performed. Then, the continuous curvilinear capsulorhexis (the capsulorhexis diameter measured with a surgical caliper was noted) and hydrodissection were completed. The phaco-chop technique was used for nuclear fragmentation at high vacuum and ultrasound power settings (350-400 mmHg and 25-45% power). It starts by embedding the phaco tip positioned bevel down into the lens nucleus and the chopper moved through the phaco tip to crack the nucleus initially. After the first crack, the chopper and the phaco tip were moved in opposite directions to separate the nuclear halves. Then, the nucleus was rotated, and half of it was impaled by the phaco tip. Chopping was performed, and the first free nuclear piece was emulsified. This process was repeated at the same energy and vacuum settings in the capsular bag as much as possible. If the moving lens pieces were emulsified closer to the anterior chamber, the changes in PP were recorded. The epinucleus was cleared with low vacuum and ultrasound power settings. Cortex removal was performed through bimanual irrigation/aspiration. After all the lens material was cleared, a sodium hyaluronate 1.4% (Protectalon) OVD was injected into the capsular bag. The same foldable one-piece hydrophobic acrylic intraocular lens (Sensar AR40, AMO, Minneapolis, USA) was inserted in each patient. Then, the OVD was removed from the anterior and posterior chambers and corneal incisions were closed by stromal hydration without any suturing. At the end of the surgery, intracameral cefuroxime 1 mg/0.1 ml was applied, and corneal incisions were checked to ensure that there was no leakage. Patients were classified by the same surgeon (EU) into three groups according to PP (Table 1).

**Table 1:** Group classification according to the plane of phacoemulsification

Groups	Changes In The Plane Of Phacoemulsification During Surgery
Group 1	75%–100% of the lens nucleus was emulsified in the capsular bag, 0%–25% of the lens nucleus was emulsified at the pupillary plane
Group 2	75%–100% of the lens nucleus was emulsified at the pupillary plane, 0%–25% of the lens nucleus was emulsified in the anterior chamber or in the capsular bag
Group 3	50%–75% of the lens nucleus was emulsified in the anterior chamber, 25%–50% of the lens nucleus was emulsified at the pupillary plane

Patients who did not meet any of the group criteria were excluded from the study. The phacoemulsification time (s), phacoemulsification power (%), and effective phacoemulsification time (EPT) were recorded. EPT was computed as follows: phacoemulsification time × phacoemulsification power/100. Total operation time was calculated as the time between the opening of the first paracentesis and the closure of corneal incisions by stromal hydration. Postoperatively, patients were treated with moxifloxacin 0.5% and dexamethasone 0.1% eye drops 6 times daily for one week, after which only dexamethasone 0.1% was instilled over three weeks with a gradually decreasing dosage.

All data was analyzed using the SPSS statistical software package, version 24.0 (IBM Corp., Armonk, NY). Parametric data were compared using analysis of variance (ANOVA), followed by the Scheffe test for post hoc comparisons, and non-parametric data were compared using the Chi-square test followed by the Mann-Whitney U test for pairwise comparisons of the groups. The associations with continuous and categoric variables were assessed using Pearson's and Spearman's-Rho bivariate correlation analyses, respectively. We also investigated the factors that could influence PP using ordinal regression analysis. The statistical significance level was set at p < 0.05.

## Results

Regarding intraoperative complications, two cases of posterior capsule ruptures, two of zonular dehiscence, six of small detachments of Descemet’s membrane, and three of radial capsulorhexis tears were noted. After excluding complicated patients, in total, 328 eyes of 328 patients were included in this study. Of these patients, 174 were male (53.0%) and 154 were female (47.0%), with an average age of 69.3 ± 8.3 (41-95) years. Of the 328 eyes, 159 (48.5%) were right and 169 (51.5%) were left. There were 153 patients (46.7%) in Group 1, 104 patients (31.7%) in Group 2, and 71 patients (21.6%) in Group 3. No postoperative complication was observed during the follow-up. The biometric and surgical characteristics of the patients are provided in Table 2.

**Table 2:** Biometric and surgical parameters of the groups *One-way ANOVA ACD: Anterior chamber depth, AL: Axial length,  CCT: Central corneal thickness, EPT: Effective phacoemulsification time, IOP: Intraocular pressure, LT: Lens thickness, NS: Nuclear sclerosis, PD: Pupil diameter with full dilatation, SD: Standard deviation

Parameters	Group 1 (n=153) Mean ± SD	Group 2 (n=104) Mean ± SD	Group 3 (n=71) Mean ± SD	P*
Age (years)	69.0 ± 8.5	70.0 ± 7.8	68.9 ± 8.6	0.284
EPT (s)	3.9 ± 2.5	4.3 ± 2.2	3.5 ± 1.5	0.093
Ultrasound power (%)	28.5 ± 6.2	28.2 ± 6.7	25.3 ± 5.8	0.010
Vacuum (mmHg)	366.4 ± 12.8	361.9 ± 12.7	366.0 ± 15.1	0.600
Total operation time (min)	13.6 ± 3.4	13.0 ± 2.9	12.7 ± 2.5	0.136
Capsulorhexis diameter (mm)	5.5 ± 0.6	5.4 ± 0.5	5.6 ± 0.4	0.511
Preoperative IOP (mmHg)	14.5 ± 3.3	14.0 ± 3.2	14.1 ± 3.6	0.592
NS grade	3.3 ± 0.7	3.4 ± 0.8	2.8 ± 0.37	< 0.001
AL (mm)	23.44 ± 0.81	23.49 ± 0.90	23.56 ± 1.05	0.718
ACD (mm)	3.29 ± 0.35	3.34 ± 0.40	3.46 ± 0.33	0.019
CCT (µm)	523.2 ± 32.7	521.5 ± 34.0	526.9 ± 32.4	0.686
LT (mm)	4.33 ± 0.42	4.35 ± 0.43	4.34 ± 0.51	0.817
PD (mm)	6.89 ± 0.97	6.90 ± 0.82	7.07 ± 0.90	0.442

Significant differences have been detected in the ultrasound power, ACD, and NS grades of the groups (the p-values were 0.010, 0.019, and < 0.001, respectively). There was no difference among the groups in other surgical, biometric, and demographic data (p > 0.05 for all). In post hoc comparisons, the ACD values of Group 3 were found to be higher than those of Group 1 (p = 0.028). In addition, the ultrasound power was less in Group 3 than in Group 1 and Group 2 (the p-values were 0.010 and 0.031, respectively). Moreover, the patients were categorized according to their NS grades (NO2-NO3-NO4-NO5 as per LOCS scale) and mean ACD values (shallow: < 3.00 mm, normal: 3.00-3.70 mm, and deep: > 3.70 mm) for a detailed evaluation of their PP distribution (Table 3).

**Table 3:** Plane of phacoemulsification distribution according to NO and ACD categories *Chi-Square test, ACD: Anterior chamber depth, NO: Nuclear opacification

Parameter	Category	Group 1	Group 2	Group 3	P*
NO	Grade 2 (n=62)	17 (11.1%)	16 (15.4%)	29 (40.8%)	0.002
	Grade 3 (n=128)	73 (47.7%)	35 (33.6%)	20 (28.2%)	
	Grade 4 (n=92)	47 (30.7%)	30 (28.9%)	15 (21.1%)	
	Grade 5 (n=46)	16 (10.5%)	23 (22.1%)	7 (9.9%)	
Total number of groups		153 (100%)	104 (100%)	71 (100%)	
ACD	Shallow (< 3.00 mm) (n=64)	37 (24.2%)	21 (20.2%)	6 (8.4%)	0.036
	Normal (3.00-3.70 mm) (n=210)	100 (65.4%)	61 (58.7%)	49 (69.1%)	
	Deep (> 3.70 mm) (n=54)	16 (10.4%)	22 (21.1%)	16 (22.5%)	
Total number of groups		153 (100%)	104 (100%)	71 (100%)	

Pairwise comparisons showed that phacoemulsification occurred at a much anterior plane in patients with NO2 cataracts than those with NO3-NO4-NO5 cataracts (the p-values were 0.001, 0.001 and 0.006, respectively). Furthermore, phacoemulsification was revealed to have been done more posteriorly in patients with shallower ACDs and more anteriorly in patients with deeper ACDs (the p-values were 0.010 and 0.031, respectively).

In the correlation analyses, the factors associated with PP were ultrasound power (r −0.149, p 0.016), ACD (r 0.139, p 0.020), and NS grade (r −0.127, p 0.028). However, partial correlation analyses controlled with the NS grade found the association between PP and ultrasound power to be statistically insignificant (r −0.113, p 0.079). No significant relationship was observed between PP and age, EPT, AL, pupil diameter, total operation time, LT, capsulorhexis diameter, CCT (r range: −0.012 to 0.87, p range: 0.141-0.836). An ordered logit model was estimated to investigate AL, ACD, NS grade, LT, and pupil diameter to predict how the PP came close to the endothelium. Together, the predictors accounted for a significant amount of variance in PP (p = 0.001). Only the NO2 grade significantly independently predicted PP (B = 1.452, p = 0.022). The ACD was detected to be a predictor close to being statistically significant (B = 0.663, p = 0.060). Overall, the model accounted for approximately 11% of the variance in PP, Nagelkerke pseudo-R2 = 0.109.

## Discussion

PP is an important factor affecting postoperative corneal edema, which is one of the major predictors for visual recovery after uncomplicated phacoemulsification surgery. Before surgery, an estimation of the factors that may affect PP will help keep the plane at the desired level during the surgery. The present study has shown that the NS grade and ACD values affect the plane at which nuclear fragments have been emulsified, whereas AL, LT, pupil or capsulorhexis diameters have no effect on this plane.

In the literature, different techniques of nuclear fragmentation have been compared to evaluate these techniques regarding safety, effective energy use, or the influence on the corneal endothelium [2,5,7-11]. However, in these studies, it was assumed that the techniques were applied perfectly as described and PP changes other than those described in the techniques that could occur during surgery were not considered. To the best of our knowledge, ours is the first study investigating the factors that can change PP while applying the same technique (phaco-chop) during the surgery. In the current study, PP could not be kept at the desired plane (behind or at the pupillary level) in 21.6% of the patients. It was observed that 40.8% of these patients have NO2-grade cataracts. Moreover, the PP was behind or at the pupillary plane in 90.6% of the patients with a shallow anterior chamber. Therefore, softer cataracts may induce the PP to move to the anterior, whereas emulsification may take place at more posterior planes in patients with shallower ACDs. In addition, bevel down position or surgeon experience can be other factors increasing the percentage of anterior plane phacoemulsification in the current study. Furthermore, the regression model of the present study explained approximately 11% of the changes in PP; therefore, there may be a lot of factors that some of these are difficult to measure objectively, affecting PP.

Previous studies have reported that supracapsular strategies such as retro-chop, phaco-out or half-moon techniques can be applied safely and effectively [2,5,8,10,12]. On the other hand, it has been suggested that the emulsification of nuclear fragments at a posterior plane is safer for the endothelium than pupillary or anterior plane phacoemulsification [11,13-16] (Table 4).

**Table 4:** Summary of the previous studies CCT: Central corneal thickness, ECL: Endothelial cell loss, LogMAR: Logarithm of the minimum angle of resolution Hwang et al. [2]; Can et al. [5]; Alio´ et al. [8]; Jeancolas et al. [10]; Perone et al. [12]; Kosrirukvongs et al. [11]; Hayashi et al. [13]; Koch et al. [14]; Uyar E [15]; Vasavada and Raj [16]

References	Sample (n)	Technique	Phaco time (second)	Corneal parameters on postoperative first days	Final BCVA	Conclusion
Hwang et al. (2016)	131 eyes of 111 patients	Stop-Chop vs Retro-Chop	31.08 vs 26.35 (p = 0.010)	increase in CCT at first week: 5% vs 4% (p > 0.05)	0.19 logMAR vs 0.19 logMAR (p > 0.05)	Retro-chop is an effective and safe method. It reduces intraoperative ultrasound energy and early postoperative corneal endothelial cell loss.
Can et al. (2008)	100 eyes of 92 patients	Half-moon supracapsular vs Stop-Chop	12.0 vs 24.0 (p = 0.001)	increase in CCT on first day: 44.9 µm vs 30.1 µm (p > 0.05)	0.09 logMAR vs 0.12 logMAR (p = 0.388)	The half-moon supracapsular technique shortened the phacoemulsification procedure and lowered phaco energy. There was no difference between techniques in reliability and functionality.
Alio´ et al. (2002)	60 eyes of 30 patients	Phacoemulsification in the anterior chamber vs Stop-Chop	63.6 vs 77.4 (p = 0.225)	increase in CCT on 1-3 days: 63.0 µm vs 47.0 µm (p > 0.05)	20/32 vs 20/32 (p = 0.692)	Phacoemulsification in the anterior chamber was as safe as endocapsular phacoemulsification using a stop-and-chop technique. This technique is fast and easier to learn than endocapsular phacoemulsification.
Jeancolas et al. (2017)	110 eyes of 96 patients	Subluxation vs Divide and Conquer	41.0 vs 57.0 (p < 0.001)	increase in CCT at first hour: 69.9 µm vs 64.4 µm (p = 0.060)	N/A	Subluxationtechnique does not result in greater CCT than the divide-and-conquer technique.
Perone et al. (2019)	2856 eyes	Supracapsular phacoemulsification (Garde-à-vous) vs Divide and Conquer	43.5 vs 64.6 (p < 0.001)	N/A	N/A	The study presented shows that the technique of subluxation described under the term of ‘‘Garde-à-vous’’ technique can be used in daily practice. The ‘‘divide and conquer’’ technique remains useful in eyes where the nucleus is excessively hard and in eyes where pupil dilation is limited.
Kosrirukvongs et al. (1997)	41 eyes	Chip and Flip vs Divide and Conquer	109.8 vs 100.2 (p = 0.724)	increase in CCT at first week: 15.5 µm, 2.8% vs 17.3 µm, 3.4% (p= 0.899)	N/A	The divide and conquer technique led to less endothelial loss and hexagonal cell change than the chip and flip technique, although at 3 months the differences were not significant.
Hayashi et al. (1996)	843 eyes of 784 patients	N/A	N/A	N/A	N/A	In conclusion, the firmness of the nucleus was the principal risk factor for endothelial injury. Careful surgical maneuvering to avoid endothelial contact with nuclear fragments will help decrease the degree of endothelial injury.
Koch et al. (1993)	59 eyes	Iris plane vs Posterior chamber phacoemulsification	78.0 vs 100.2 (p = N/A)	ECL at week 16 in Healon subgroup: 13.8% vs 0.6% (p < 0.03)	N/A	Posterior-chamber phacoemulsification results in less endothelial injury than iris-plane phacoemulsification, particularly when Healon is used.
Uyar E (2022)	232 eyes of 232 patients	Phaco-Chop Three plane of phacoemulsification: relatively anterior- iris plane- capsular bag	14.8- 14.1 -14.0 (p = 0.862)	increase in CCT on first day: 81.4 µm - 60.4 µm -42.2 µm (p < 0.001)	N/A	More anterior phacoemulsification planes than the capsular bag caused a higher CCT increase postoperatively.
Vasavada and Raj (2003)	N/A	Step-down technique	N/A	N/A	N/A	As the procedure progresses, posterior capsule exposure is a concern. Often, the surgeon subconsciously brings the phaco probe anteriorly. However, the plane at which the fragments are removed is crucial to a successful surgical outcome. Removal of fragments in the posterior plane is preferred. This can be safely achieved by the use of the step-down technique.

A recent study has also found that more anterior planes than the capsular bag increase corneal edema and that this edema can last for more than a week [15]. Furthermore, studies assessing the effect of the phaco tip position on endothelial cell loss have discussed which tip position keeps the ultrasound energy far from the endothelium and brings the PP closer to the capsular bag [17,18]. Some authors claimed the bevel up position has fewer negative effects on the corneal endothelium, while a contrary bevel down position has been suggested by others [17,19]. The consensus among these studies is that increasing the distance between the phaco tip and the endothelium is an important factor in reducing endothelial cell damage [17-19]. Considering all of the above, although some supracapsular techniques have been developed as safe and effective strategies, the common opinion of keeping the phaco probe away from the endothelium maintains a key role in protecting endothelial cells during phacoemulsification surgery [3,11,14-19].

Limitations

The present study has some limitations. First, all of the quantitative or qualitative factors that may affect PP, including the different positions of phaco tip, intraoperative pupil changes, patient compliance, surgeon comfort, surgeon skill, and particularly surgeon experience, could not be evaluated. Second, only one technique has been assessed regarding intraoperative changes in PP. Third, the PP and patient groups have been determined by the same surgeon’s visual judgment. The current study has certain advantages as well. The eye-related factors affecting PP have been evaluated objectively with the contribution of standardized variables, i.e., surgeon, the technique of nuclear fragmentation, and the phaco machine. In addition, the method of the present study can adequately represent cataract surgery practice because changes in PP level were evaluated during live surgery.

## Conclusions

In conclusion, in phacoemulsification surgery, a balance should be maintained, keeping the plane far enough from the cornea and the posterior capsule. And, the NS grade and ACD values can affect this balance and changes in PP during surgery. Softer cataracts may increase the fear of posterior capsule rent when the phaco-chop technique is used in the capsular bag; the surgeon may, therefore, bring the phaco probe anterior to the pupillary plane subconsciously. On the other side of this balance, shallow anterior chambers or high-grade cataracts may increase the fear of endothelial damage; thus, the surgeon may bring the phaco probe posterior to the pupillary plane. Similarly, a deep anterior chamber reassures the surgeon that it is far enough from the endothelium, thereby causing the surgeon to hold the phaco probe anterior to the pupillary plane. Therefore, to stay away from endothelium, energy and vacuum settings can be decreased as the phaco tip approaches the deeper plane, especially in low-grade cataracts, and more effort can be put in for patients with deep ACD when the phaco-chop technique is performed in the capsular bag. In addition, it is important maintaining a formed, non-fluctuated anterior chamber with the contribution of reduced wound leakage, appropriate settings of vacuum, aspiration rate, and bottle height to keep the plane of phacoemulsification at a desired level in the chamber. Further studies are needed to evaluate the various factors that can affect the PP while applying different nuclear fragmentation techniques in the future.
